# Ingestion of Bell Clappers by a Shaman in Jumla, Nepal: A Case Report

**DOI:** 10.31729/jnma.4055

**Published:** 2019-02-28

**Authors:** Niresh Thapa, Subi Basnyat, Muna Maharjan

**Affiliations:** 1 Karnali Academy of Health Sciences, Jumla, Nepal; 2 Second Clinical College of Wuhan University, Wuhan, P. R. China; 3 HOPE School of Nursing, Zhongnan Hospital of Wuhan University, Wuhan, Hubei, China

**Keywords:** *foreign bodies*, *Nepal*, *shaman*

## Abstract

Accidental foreign body ingestion is a common problem encountered in Emergency. Deliberate foreign body ingestion may result due to an act of insanity or an act of daring. A shaman locally known as Dhami was brought to Emergency with the history of ingestion of bell clappers. He denied the history of psychiatric illness or substance abuse. On physical examination, there were signs of peritonitis. Laparotomy was done to remove the foreign bodies. Post-operative period was uneventful. Apart from the surgical intervention, psychological counselling was given to him. This is a rare interesting case due to the fact that the 15 cm long foreign bodies passing all the way through without significant injury and finally causing obstruction in ileocecal junction and perforation in the distal ileum.

## INTRODUCTION

Accidental foreign body (FB) ingestion is a common problem encountered in Emergency, but it can also occur in a patient with a psychiatric illness or other issues.^1^ Deliberate FB ingestion may result due to an act of insanity.^2^ Here we report a rare case of ‘shaman’ (or ‘Dhami’ in the Nepali language). ‘Shaman’ is referred to a person, especially among certain tribal peoples, who acts as an intermediary between the natural and supernatural worlds using magic to cure illness, foretell the future, control spiritual forces etc.^3^

## CASE REPORT

A 47 year's man was brought to the Emergency Department of Karnali Academy of Health Sciences (KAHS) with chief complains of pain abdomen associated with several episodes of vomiting and high-grade fever for 3 days. He used to be a ‘Shaman’ in the village. He had given the history of ingesting 4-5 bell clappers and alcohol 60 days earlier on the special occasion of full moon day. Moreover, according to him and his visitor, he has been ingesting the same (altogether 305) on different occasions for 16 years. There was no history of trauma, denied the past history of psychiatric illness or taking any medication. On physical examination, he was ill-looking and dehydrated; blood pressure was 130/90 mm of mercury, the temperature was 38^D^Cand other vitals were normal. There was generalized abdomen tenderness, and rebound tenderness in right iliac fossa. Another systemic examination was normal. We made a working diagnosis of acute abdomen likelyviscous organ perforation due to the foreign body.

**Figure 1.**
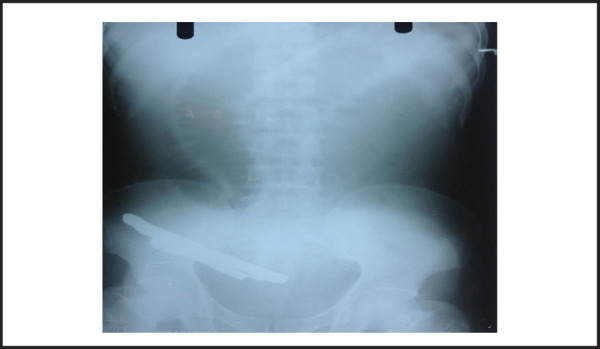
X-ray abdomen: the radiopaque shadow in the pelvic cavity and centrally multiple dilated bowel loops were noted, suggesting foreign body and likely bowel obstruction.

Investigations: WBC-16.9x10^9^ per L, Neutrophil-89%, Lymphocyte-10%, Haemoglobin-15 gm/dl; Urine, blood sugar, renal function test, andserology tests were all normal. X-ray abdomen: the radiopaque shadow in the pelvic cavity and centrally multiple dilated bowel loops were noted, suggesting foreign body and likely bowel obstruction (Figure 1).

**Figure 2.**
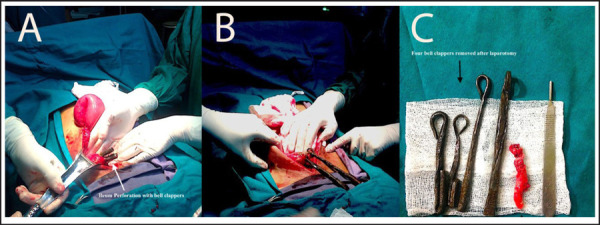
A. Ileum perforation with bell clappers. B. Two bell clappers removed during surgery. C. Foreign bodies measuring 15 cm and 10 cm long each two (four Bell clappers) and appendix.

**Figure 3.**
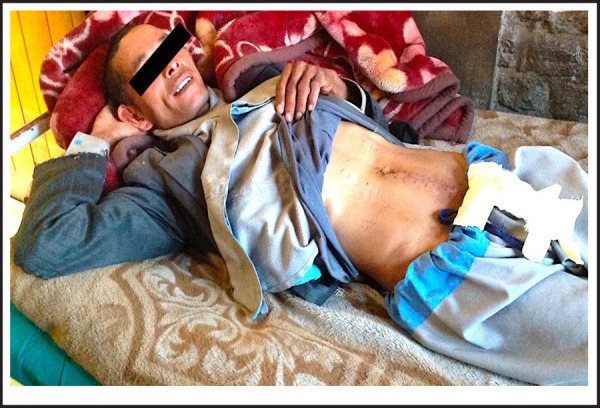
The patient on the 10^th^ postoperative day after suture removal.

Laparotomy was performed. Intra-operative findings were: perforated distal ileum with minimal faecal particle contamination, swollen inflamed appendix and obstructed ileocecal junction by FB. FB measuring 15 cm and 10 cm long each two (four Bell clappers) were removed (Figure 2), bowl perforation site was closed in two layers and appendectomy was done followed by a normal saline wash. Post-operative period was uneventful. The patient was discharged followed by suture removal on the 10th postoperative day (Figure 3). Psychiatric counselling was given to the patient. On follow-up in 1 and 6 months, he was physically fine and mentally sound.

## DISCUSSION

Foreign body ingestion is a common gastrointestinal tract emergency. Deliberate ingestion may result from an act of insanity or dare.^2^ Bell is regarded as the holy object in a temple, so the shaman who ingests bell clappers was believed to be near to the god and people trust him more. Retrospectively, this shaman (patient) analysed and concluded this incident while he was in ER. He has been ingesting bell clappers for years without any complications which he successfully ‘digested’. But this time it was complicated because of the alcohol he consumed and the ‘touch’ by lower caste during his pray. This case has indicated that even in the modern age of Nepal, there is shaman active in some parts of Nepal practising as a traditional healer. They not only harm their believers but sometimes harm themselves. It is occasionally heard that shaman asking their believers to walk on burning coal or to swallow boiling oil in order to heal the illness caused by ghosts or witch. Shaman is especially active in the region with low socioeconomic status and higher illiteracy rate. The presence of shaman may also be due to the absence of good accessible health care facility and widespread superstitious belief in the society. There is a report that many individuals with deliberate FB ingestion are usually suffered from serious psychiatric and social problems which include depression, alcoholism and imprisonment.^4,5^ Therefore, it is necessary to have the health awareness program in the community, to counsel the shaman by needful psychiatric intervention and ultimately having a good functioning health system.

Coin is the common FB ingestion among paediatric population. Other foreign bodies ingestion might be fish or chicken bones, batteries, and artificial dentures etc. Complications of the FB ingestion include ulceration, lacerations, perforation, intestinal obstruction, fistula formation etc. Most of FB pass spontaneously without causing significant complication but less than 1% of FB causes a perforation in acute angulation site but majority happens around the ileocecal area.^4^ It is reported that FBs thicker than 2 cm and longer than 5 cm are unlikely to pass the pylorus spontaneously and usually over 10 cm long fails to traverse the duodenal sweep.^2^ This is a rare interesting case due to the fact that the 15 cm long FBs passed through pylorus-duodenum without significant injury and finally causing obstruction in ileocecal junction and perforation in distal ileum.
